# Raising Suspicion of Thrombotic Thrombocytopenic Purpura in a Patient With a Pre-existing Autoimmune Disease

**DOI:** 10.7759/cureus.77623

**Published:** 2025-01-18

**Authors:** Naveena L Murugan, Richard Mensah, Atul Singh

**Affiliations:** 1 Internal Medicine, West Virginia University School of Medicine, Charleston, USA; 2 Internal Medicine, Charleston Area Medical Center, Charleston, USA

**Keywords:** adamts 13, autoimmune connective tissue disease, plasmic score, rheumatoid arthritis, sjogren's syndrome, systemic lupus erythematosus, thrombotic thrombocytopenic purpura (ttp)-like syndrome

## Abstract

Thrombotic thrombocytopenic purpura (TTP) is a rare form of microangiopathic hemolytic anemia with high rates of mortality without treatment. Common risk factors for TTP include immunosuppression, pregnancy, and female gender. However, several case reports show that TTP may have an association with autoimmune conditions such as Sjögren's syndrome (SS), rheumatoid arthritis (RA), and systemic lupus erythematosus (SLE). We present a similar case of a 41-year-old female with a past medical history of RA, SLE, and SS, who arrived at the hospital with hematuria, flank pain, slow speech, and altered mental status. Based on her presentation, there were concerns for TTP, hemolytic uremic syndrome, glomerulonephritis, or sepsis secondary to urinary tract infection. After diagnosis was narrowed to TTP, treatment was initiated for TTP with plasmapheresis, methylprednisolone, and rituximab infusions prior to receiving diagnostic confirmation due to high clinical suspicion. Upon further workup, her autoimmune and immunology panels returned several days post-admission with low ADAMTS13 activity, confirming the TTP diagnosis. Her autoimmune conditions were also confirmed for SS, RA, and SLE based on positive serology for anti-SSA/Ro antibodies, anti-CCP antibodies, and speckled ANA, respectively. With treatment, the platelet counts increased, and the symptoms present at admission resolved over a prolonged hospital course. Initiating treatment for TTP should be based on findings of clinical and routine laboratory testing rather than confirmatory test results due to the delay in receiving results, such as the ADAMTS13 level. In patients with a history of autoimmune disease, the association between TTP and autoimmune diseases can help formulate a clinical diagnosis of TTP early in the hospital course, allowing for treatment initiation and decreased mortality.

## Introduction

Acquired thrombotic thrombocytopenic purpura (TTP) occurs when anti-ADAMTS13 autoantibodies form and decrease serum levels of ADAMTS13, a protease that cleaves von Willebrand factor [1]. Decreased levels of ADAMTS13 result in a prothrombotic environment due to elevated levels of von Willebrand factor multimers that are left uncleaved and active [1]. This results in the formation of microthrombi in the blood vessels, leading to platelet depletion, schistocyte formation, and end-organ damage. As a result, patients present with fever, anemia, thrombocytopenia, renal symptoms, and neurological symptoms [1]. Triggers for anti-ADAMTS13 antibody formation include pregnancy, human immunodeficiency virus infection, and immunosuppressive and antiplatelet medications [1]. In addition, TTP is reported to have an association with autoimmune diseases such as SS and SLE [2]. TTP is diagnosed by measuring the activity level of ADAMTS13, and the risk of TTP can be predicted using the PLASMIC score. The laboratory analysis of ADAMTS13 activity is not commonly available at all healthcare centers, so it is often analyzed at outlying facilities, which causes results to be delayed by several days. Furthermore, untreated TTP has a 90% mortality rate, so it is important to promptly diagnose TTP and initiate treatment [3].

## Case presentation

A 41-year-old female with a past medical history of Sjögren's syndrome (SS), systemic lupus erythematosus (SLE), and rheumatoid arthritis (RA) presented to the hospital with a two-day history of classic TTP symptoms of hematuria, flank pain, slow speech, somnolence, and altered mental status. A physical exam revealed that the patient appeared acutely ill and somnolent. Laboratory analysis showed the presence of schistocytes, elevated inflammatory markers, acute kidney injury due to elevated BUN and creatinine, thrombosis due to elevated D-dimer and low platelet count, hemolysis due to elevated bilirubin and lactate dehydrogenase, and decreased haptoglobin (Table 1). End-organ inflammation of the heart and kidneys was suspected due to elevated creatine kinase, troponin I, and inflammation of the renal fascia with abnormal urinalysis (Table 2). Based on patient presentation, differential diagnoses of TTP, hemolytic uremic syndrome, glomerulonephritis, or sepsis secondary to urinary tract infection were considered. Due to concern for sepsis, blood cultures were taken, and the patient was given broad-spectrum antibiotics, cefepime and Zyvox, which were later discontinued. The PLASMIC score was calculated to be 5, suggesting an intermediate risk for TTP.

**Table 1:** Serum laboratory values indicating acute kidney injury, hemolysis, thrombosis, and inflammation at admission H: high

Acute kidney injury	Serum laboratory values
Blood urea nitrogen	52 mg/dL (H)
Creatinine	3.2 mg/dL (H)
Hemolysis	-
Hemoglobin	14.9 g/dL
Hematocrit	42.9%
Mean corpuscular volume	93.8 fL
Total bilirubin	2.9 mg/dL (H)
Direct bilirubin	0.36 mg/dL (H)
Haptoglobin	<30 mg/dL (L)
Lactate dehydrogenase	2643 unit/L (H)
Thrombosis	-
Platelets	12 x 10^3^/mcL (L)
D-dimer	19.23 mg/L (H)
International normalized ratio	1.15
Inflammation	-
Erythrocyte sedimentation rate	56 mm/hr (H)
C-reactive protein	110.5 mg/L (H)
Procalcitonin	1.59 ng/mL (H)

**Table 2:** Serum laboratory values indicating end-organ inflammation to the heart and kidneys HPF: high power field, LPF: low power field, H: high

Cardiac damage		Serum laboratory values
	Creatine kinase	253 unit/L (H)
	Serial troponin I	331, 449…2784 pg/mL (H)
Kidney damage (urinalysis)	Color	Red
	Specific gravity	1.036 (H)
	Protein	100 mg/dL (H)
	Red cells	>182/HPF (H)
	White cells	10/HPF (H)
	Blood	Large
	Squamous epithelial cells	35/HPF (H)
	Hyaline casts	11/LPF (H)

While awaiting the results of the autoimmune and immunology panel for laboratory diagnosis, TTP treatment was initiated based on clinical suspicion. After ruling out the infection, the patient received intravenous normal saline and methylprednisolone 125 mg every six hours and underwent plasmapheresis with 100% fresh frozen plasma replacement. The patient was then switched to methylprednisolone 60 mg daily. Four days after admission and treatment initiation, the autoimmune and immunology panels returned and reported low ADAMTS13 activity, confirming the diagnosis of TTP. The panel also verified the patient’s autoimmune history for SS, RA, and SLE based on positive serology for anti-SSA/Ro antibodies, anti-CCP antibodies, and speckled ANA, respectively.

**Figure 1:**
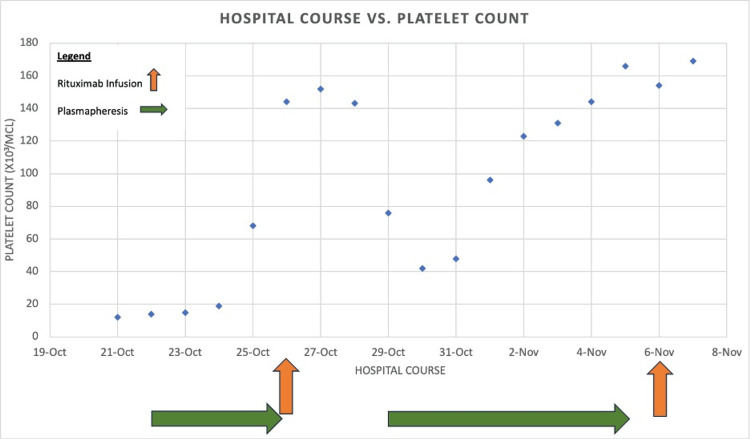
Trend of patient’s platelet count during plasmapheresis and rituximab treatment. Plasmapheresis was initiated on 10/22 after schistocytes were found on peripheral smear. Rituximab infusions were done on 10/26 and 11/6

Due to the confirmed diagnosis of TTP, treatment was advanced to rituximab 375 mg/M2 four times weekly. Throughout the hospital course, the platelet counts fluctuated but eventually improved from the baseline of 12 x 103/mcL to 169 x 103/mcL (Figure 1). The ADAMTS13 levels normalized 17 days after her initial admission to the hospital. Caplacizumab was planned to be administered in the case of future relapse.

## Discussion

Noting an association between autoimmune conditions and TTP can help raise clinical suspicion for TTP when a patient with autoimmune disease presents with fever, anemia, thrombocytopenia, and renal and neurological symptoms [4-7]. Literature review shows that SS is associated with TTP, and in many cases, TTP onset occurs after one has been diagnosed with SS, suggesting that SS may induce TTP [5]. In the pathophysiology of SS, many autoantibodies are produced, such as anti-SSA and anti-SSB, and some newly discovered autoantibodies [8]. Of these novel autoantibodies, it is possible that the anti-ADAMTS13 antibody is formed, resulting in TTP manifestation later in the SS disease course [5].

A study conducted in Japan examined 422 cases of TTP, of which 25.6% of individuals had a connective tissue disease, of which SLE was the most predominant [7]. In addition, a case report in 1999 described the development of TTP in a patient with RA [6]. These reports suggest an association between TTP and autoimmune connective tissue diseases.

The PLASMIC score can be used to identify the likelihood of TTP based on presenting symptoms [1]. This patient has a PLASMIC score of 5, suggesting an intermediate TTP risk (Table 3). This suggests that the sensitivity of the PLASMIC score may be lower in patients with autoimmune connective tissue diseases. This tool may be more clinically representative by adding the past medical history of autoimmune disease (SS, RA, and SLE) as one of the diagnostic likelihood criteria. This apparent association between autoimmune disease and TTP may make the screening tool more discriminative.

**Table 3:** Components of the PLASMIC score, where one point is assigned for each applicable item * reticulocyte count >2.5%, undetectable haptoglobin, or indirect bilirubin >2 mg/dL [1]

PLASMIC score criteria	This patient
Platelet count <30 x 10^3^/mcL	1
Hemolysis*	1
Mean corpuscular volume <90 fL	0
International normalized ratio <1.5	1
Creatinine	0
Absence of cancer	1
Absence of solid organ or stem cell transplant	1
Total	5

TTP diagnosis based on specialized laboratory testing of ADAMTS13 levels takes several days to complete, as most hospitals do not have ADAMTS13 testing capabilities on-site. The high mortality rates of untreated TTP should motivate clinicians to initiate TTP treatment based on clinical presentation, peripheral smear, and standard laboratory testing alone. By using tools such as the PLASMIC score and recognizing one’s past medical history of SS, RA, or SLE, there can be a higher index of suspicion for TTP. This clinical diagnosis of TTP will allow treatment to be initiated early, which will help reduce associated mortality and complications.

## Conclusions

Untreated TTP has high rates of mortality, and confirmatory laboratory diagnosis of TTP is difficult due to the prolonged wait time for ADAMTS13 test results. Healthcare professionals must rely on the clinical diagnosis of TTP via clinical presentation and standard laboratory testing to initiate treatment early. The association of TTP with autoimmune diseases such as SS, RA, and SLE has been well-reported in several case reports. It may be an additional factor in raising clinical suspicion of TTP. Further investigation of this association will help clinicians gain more confidence in the clinical diagnosis of TTP, accelerating the time to diagnosis and treatment and thereby improving outcomes.
